# Autism Spectrum Disorder and IQ – A Complex Interplay

**DOI:** 10.3389/fpsyt.2022.856084

**Published:** 2022-04-18

**Authors:** Nicole Wolff, Sanna Stroth, Inge Kamp-Becker, Stefan Roepke, Veit Roessner

**Affiliations:** ^1^Department of Child and Adolescent Psychiatry, Medical Faculty of the Technische Universität (TU) Dresden, Dresden, Germany; ^2^Department of Child and Adolescent Psychiatry, Psychosomatics and Psychotherapy, Philipps University of Marburg, Marburg, Germany; ^3^Department of Psychiatry, Charité – Universitätsmedizin Berlin, Berlin, Germany

**Keywords:** autism spectrum disorder (ASD), ADOS, IQ, diagnostics, heterogeneity

## Abstract

Autism spectrum disorder (ASD) is characterized as a very heterogeneous child-onset disorder, whose heterogeneity is partly determined by differences in intelligence quotient (IQ). Older epidemiological studies suggested that the IQ-related spectrum tends to be skewed to the left, i.e., a larger proportion of individuals with ASD have below average intelligence, while only few individuals with ASD may have an IQ above average. This picture changed over time with broadening the spectrum view. Within the present perspective article, we discuss discrepancies in IQ profiles between epidemiological and clinical studies and identify potential underlying aspects, for example, the influence of external factors such as sample biases or differences in availability of autism health services. Additionally, we discuss the validity and reciprocal influences of ASD diagnostics and IQ measurement. We put the impact of these factors for diagnostic as well as care and support situations of patients into perspective and want to encourage further research to contribute to the conceptualization of “autism” more comprehensively including the IQ as well as to examine broader (life) circumstances, interacting factors and diagnostic requirements of given diagnoses in childhood as compared to adulthood.

## Introduction

Autism spectrum disorder (ASD) is characterized as a very heterogeneous child-onset disorder. The clinical picture including the severity of the core deficits of ASD varies significantly among individuals leading to individually different degrees of functional qualities in ASD as well as to difficulties in correctly recognizing and diagnosing ASD (1).

**FIGURE 1:**
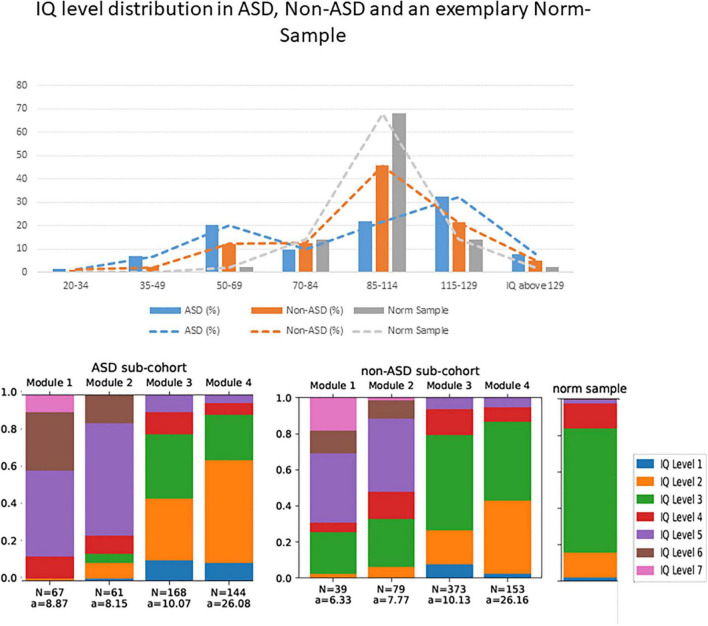
*Upper part:* The IQ level distribution of the sample described in Wolff et al. (2022) as well as an exemplary norm sample. The *y*-axis shows percentages, and the *x*-axis shows the IQ values, summarized from IQ level 7 – 1 (left to right) according to the multiaxial system (20, 21). *Lower part: N* = number of included individuals per ADOS module and sub-cohort, a = Mean age of the included individuals in years. The IQ level distribution shows the categorization according to the psychiatric multiaxial schema (20, 21). Within this scheme, individuals can be categorized with respect to axis 3, “intellectual level” according to the following classification: IQ level 1 = IQ > 129, IQ level 2 = IQ = 115 – 129, IQ level 3 = IQ = 85 – 114, IQ level 4 = IQ = 70 – 84, IQ level 5 = IQ = 50 – 69, IQ level 6 = IQ = 25 – 49, IQ level 7 = IQ = 20 – 34, IQ level 8 (not depicted here, since we had no participants which are labeled with IQ level 8) = IQ < 20. We also show the IQ level distribution in a norming sample.

One key aspect of the heterogeneity of ASD symptomatology appears to be the heterogeneity in intelligence quotient (IQ) (2). For example, Fombone (3) reported of 20 epidemiological studies of ASD, published from 1966 to 2001 and deduced that the median percentage of individuals with ASD and cognitive impairment (IQ < 70) ranged from 40 to 100% (mean 70%). This indication is also in line with statements in the current German and British ASD diagnostic guidelines (4, 5). In the early 2000s another large epidemiological study reported that an IQ < 70 was observed in only 50% of children with ASD (6), while a more recent epidemiological study (7) reported a further decline toward an amount of 31% of children with ASD, that were classified in the range of cognitive impairment (IQ < 70). The latter study further reported that 25% of children with ASD were in the borderline range (IQ 71–85), and 44% had IQ scores in the average to above average range (IQ ≥ 85). Unfortunately, epidemiological studies report about their individuals with ASD with above average IQ less accurately or rather insufficiently, i.e., either offer no information (8) or summarize the percentages of the group with mean and the group with above average IQ (7, 9). Compared to the accuracy of the presentation of IQ data in epidemiological samples, data in clinical studies are often even less precise. Nevertheless, one clinical study with slightly more precise information on IQ in ASD reported that 23% of the participants had an IQ < 85, while 45% had an average IQ, and 32% had an IQ above average (10). Another clinical study divided the children with ASD in a group with an IQ < 80 (32%, mean composite IQ of 66 ± 11) and IQ > 80 (68%, mean composite IQ of 99 ± 13) (11). These numbers deviate from those in epidemiological studies, as they report of substantially more individuals with above average IQ and fewer individuals with below average IQ including ID. Finally, we recently observed in a larger sample of patients, who presented in specialized outpatient clinics for ASD, a bimodal IQ distribution within ASD individuals [38.2% below average intelligence (i.e., IQ < 85), 40% with above average intelligence (IQ > 115) and 21.8% with an average intelligence (IQ between 85 and 115), see Figure 1]. In addition, we could show that only a third of ASD individuals, included in these analyses, are on average under the age of ten when receiving their ASD diagnosis, while another third of ASD individuals are on average older than 20 years when they received an ASD diagnosis. However, these cross-sectional clinical findings are observations, which require further clarification.

To sum up this first paragraph as one aspect of our perspective article, both, epidemiological and clinical data on the IQ distribution in ASD draw a picture in which the number of autistic individuals with intellectual disability seemed to have decreased from 70 to 50% down to 30% during the past 50 years. Why are we observing such a decline of individuals with ASD with intellectual disability across time?

## Heterogeneity as Effigy of an Altered Autism Spectrum Disorder Taxonomy

At first, two different conceptualizations of autism [see also descriptions of Kanner (12) and Asperger (13)] with different prototypic functional qualities and cognitive abilities were described. Both contributed to our understanding of the autism spectrum today. Since then, several further descriptions and definitions of the disorder developed: The term “infantile autism” first appeared as a diagnostic label in the Diagnostic and Statistical Manual of Mental Disorders, Third Edition (DSM-III, published in 1990). Diagnostic criteria for the Asperger syndrome and other subtypes [autistic disorder, Rett disorder, childhood disintegrative disorder, and pervasive developmental disorder not otherwise specified (PDD-NOS)] first appeared in the DSM-IV (published in 1994). Based on the DSM-IV and years of research, the DSM-5 (published in 2013) now describes a shift away in taxonomy from the categorical approach with specific subtypes to the conceptualization of autism as a spectrum (ASD). This shift away from clearly separable specific subtypes occurred as the consequence of failure to describe the heterogeneity of autism in empirically defined subcategories (14–16). Broadening of the autism concept was associated with a significant increase in research of “high functioning autism” [a term used for ASD individuals with an IQ of ≥70, i.e., *not* IQ above average (17)], reflected by substantially more publications with this keyword compared to those on “low functioning autism” (18). With the assumption that ASD is a dimensional disorder, a continuum of ASD symptoms was conceptualized and “autistic traits” can be found and examined in any psychiatric or neurodevelopmental disorder as well as in individuals without any disorder (19). Altogether, this contributes to a steady increase in heterogeneity of ASD and may thus explain a potentially increase of reported autistic individuals with an average or above average IQ. In addition, since older compared to more recent epidemiological studies did not include individuals with Asperger syndrome, the mentioned discrepancies in IQ data for individuals with ASD may also be attributed to developments in taxonomy.

## A Variegated Picture as a Consequence of Heterogeneity in Autism Spectrum Disorder Studies (E.G., in Methods or Aims) and Changes in Care Situation

Interpreting and summarizing data on IQ distribution in ASD from epidemiological and clinical studies together appears difficult due to several reasons. At first, heterogeneous and sometimes insufficient depth of detail of information limits interpretation and comparability. Further, differences in sex, ethnicity (7, 20), and age (21, 22) in epidemiological and clinical studies on ASD limit interpretation and comparability because IQ distribution varies by these factors. Also, the discrepancies of reported IQ distributions between epidemiological and clinical studies on ASD could be the consequence of referral biases resulting from a systematic selection of individuals, particularly those presenting themselves in tertiary-care centers for ASD due to higher severity of core symptoms, refractoriness to regular treatment, etc. (23–25). Similarly, the Berksonian bias as the higher mathematical chance to be referred (referral rate for disorder A+ referral rate for disorder B) further increases the differences between epidemiological and clinical samples (26). For example, a study from a German tertiary center reported that on average, parents visited 3.4 different professionals until their child received an ASD diagnosis (27), resulting in a specific sample of individuals with ASD in tertiary centers due to an overrepresentation of children of parents who take on this long-lasting diagnostic process because their child is particularly severely impaired in daily functioning or suffers from very burdensome social interaction difficulties. In addition, in ASD a high persistence of symptoms (28) and only mild to moderate treatment effects with partial symptom reduction (29) are common and this increases the search for help in tertiary centers. For the sake of completeness, it has to be generally stated that many clinical and/or experimental studies on ASD – as on other disorders – excluded individuals with an IQ < 70 due to compliance problems with the consequence of underrepresentation of this “ASD subgroup” [e.g., (30–32)].

Curiously, in the last years case constellations emerged, in which parents press for the diagnosis although their child is not severely impaired. At first, receiving an ASD diagnosis may be seen as a chance to get faster, more and/or higher-quality medical and social support. Particularly children with intellectual disability could thereby receive better support (e.g., inclusion aids, support systems, school assistance, parent training as well as disadvantage compensation) than without an ASD diagnosis. Thus, it has to be considered that individuals who receive an ASD diagnosis may receive it instead of or in addition to another diagnosis, against better knowledge of the diagnosing expert, but for family support purposes (32). At second, ASD may be perceived as less stigmatizing than other psychiatric diagnoses, like intellectual disability or ADHD (27, 32, 33).

Therefore, the broadening of the diagnostic concept of ASD, differences (in sample composition etc.) between ASD studies as well as changes of the care situation may explain the heterogeneity including the shift in IQ distribution over years (3).

## Difficulties in Terms of Validity: IQ Measurement, Autism Spectrum Disorder Measurement and Their Interplay

If these mentioned aspects were not already difficult enough, problems in terms of the validity of IQ measurement and/or ASD diagnostics as well as the interplay between both further complicates interpretation and summary of data.

There is consensus that intelligence (e.g., IQ > 70) is associated with better outcomes in life and should be optimally measured for clinical and scientific reasons with a well-established, standardized IQ test that – following the Cattell–Horn–Carroll theory – includes several subtests to measure as many of the broad abilities as possible (34). In individuals with ASD, such differentiated and full-scale IQ tests often show a heterogeneous picture with high values in some subtests and low ones in others [e.g., individuals with ASD often show decreased processing speed (35) but increased fluid reasoning or visual spatial processing (36)]. Importantly, it is relevant which IQ test is applied. For example, tests that measure a general level of the IQ, like the Multiple-Choice Vocabulary (MCV) Test, seem to be less valid as compared to full-scale IQ tests, like Wechsler tests, since MCV tests are not suited to estimate the premorbid IQ level (37). Although a high IQ is generally not considered to be problematic, it could possibly not represent the individual’s “real” intelligence, because the IQ test records solely a specific insular talent of the individual with ASD or the full scale IQ may be biased by the selection of subtasks coincidentally matching with the individual’s insular talent. On the other hand, the result of the IQ test could also be underestimating, considering the fact that individuals have to perform often in restricted time spans, and could indicate that processing speed might be impaired. In addition, there is a large gap between IQ and adaptive behaviors in real life, suggesting that estimates of IQ “alone are an imprecise proxy for functional abilities when diagnosing autism spectrum disorder, particularly for those without intellectual disability” [(10), p. 221). Finally, most IQ tests have limited precision in particular groups, especially if individuals are severely impaired (38).

Thus, one may even wonder whether the IQ is a meaningful construct to measure in ASD at all. Possibly, other abilities like “the quality of social communication” might be an approach to stratify ASD [see also the approach of Bishop et al. who propose that measuring different types of social-communication impairments might be relevant for differential diagnosis in ASD (39)] or to differentiate between ASD and non-ASD.

Considering the validity of ASD diagnostic instruments it has to be taken into account that intelligence has an influence on (a) the quality or quantity of symptoms shown by the individual (40) and/or (b) on the extent to which the person is able to understand and answer the questions and/or (c) to anticipate which behavior might be the (socially) desired in the respective situation. This leads up to the assumption, that knowledge of the IQ distribution of respective population samples is a prerequisite for the conception, evaluation and implementation of specific and valid diagnostic tools, therapeutic interventions as well as (experimental) research. Based on a previous finding, we further think that the influence of intelligence on existing and well established ASD diagnostic tools should be considered more explicitly. In this vein, we observed recently, that with respect to the cut-off exceedance of the Autism Diagnostic Observation Schedule-2 [ADOS-2 (41)], individuals with below average IQ are significantly over classified (=false positives), while individuals with above average IQ are significantly under classified or misclassified (=false negatives) in regard to an ASD diagnosis [Wolff et al. (42)]. The misclassification of individuals with above average IQ leads to further questions. Frequently it is argued, that these false negatives may be a result of the development of compensation strategies (39) also in combination with masking, both together called camouflaging (38). However, one can also question, whether ASD symptoms might change during development.

## Heterogneity Because of Developmental Trajectories

Only few studies (25, 35–37) examined developmental trajectories in ASD longitudinally and to the best of our knowledge only one, analyzed both expert- and parent-report data (43) to assess *whether* and *how* ASD symptoms might change during development. The authors observed that developmental trajectories of children with ASD are described by both, continuity (expert data) and change (parent data). Within another longitudinal study on the ADOS (expert data) it was reported that in 80% of the investigated children their ASD core features were relatively stable over a period of 8–12 years across different ages and levels of functioning (28); only in a small number of individuals with ASD symptom severity increased or decreased over time. Age, gender, race, and non-verbal IQ did not predict changes in symptom severity. Nonetheless, developmental trajectories of symptom severity and adaptive functioning are quite heterogeneous in ASD (44). They seem to depend on (a) the type of symptom (b) the intellectual level as well as (c) the age of the individual at the time of the initial diagnosis. For example, social interactions appear to be more prone to developmental changes than repetitive behaviors (45), but see Lord et al. (46) who observed that repetitive sensory motor behaviors in an intellectually able young child with ASD may have a different meaning than the same behavior that persists in an older child with or without significant delays. Hence, even severely impaired children may improve substantially, so that they may enter adolescence with severity scores that are comparable to high functioning children (47). In line with these studies, a more recent longitudinal study (48) reported that verbal adults with ASD showed significant reductions in the prevalence of several symptoms exhibited during childhood. The authors concluded that improvements suggest that symptoms indicative of ASD in young children may no longer be diagnostic markers in adolescents and adults. However, they only reported results from the Autism Diagnostic Interview – Revised [ADI-R (49), parent data] conducted several times, which is a parents or caregiver report and may possibly be biased and less objective as compared to observations of externals/experts. In line with this assumption, studies that investigated parental reports (ADI-R data) showed some improvements in ASD behaviors, especially if the individuals are high-functioning (48, 50). Others add to this finding, that continuity in the expression of symptoms is higher when individuals are low functioning and that the ability to change (i.e., to improve) is higher when individuals are high-functioning. Further, parents (documented *via* the ADI-R) tend to see more improvements over the years of development than experts (documented *via* the ADOS). These deviations were explained by the suggestions that parents may (a) tend to remember their children as more impaired earlier and consequently to see more improvements over the years or (b) habituate to their children’s behavior (43). However, most individuals with ASD would be re-diagnosed if they were diagnosed between the ages of 2 and 5 and retested in adolescence/adulthood (43).

## Profound Developmental Disorder and Diagnosis in Adulthood

It is still a matter of debate whether individuals, who were seen in a psychiatric practice or specialized outpatient clinic during childhood and adolescence, may have been diagnosed correctly with another disorder, for example ADHD, at that time and later, e.g., in adulthood, with ASD. In this context, also the sample characteristic *with* or *without* suspicion of having ASD seems to be of importance and increases heterogeneity of study findings. Although both diagnoses could not be given concomitant for a long time, studies showed that about 80% of the children with ASD report ADHD related problems and vice versa (45, 46). Of course, disorder specific diagnostic instruments are designed to be sensitive and to detect the particular disorder, but (per definition) not another one. In addition, overarching test instruments (e.g., the CBCL) have also been observed to not be suitable for the identification of ASD (51).

In addition to the mentioned longitudinal studies on the course of ASD symptoms there are only few studies that focused on adults first diagnosed with ASD. Most of them did not investigate *if* and *when* other or co-existing psychiatric diagnoses were given, i.e., in childhood or relatively shortly before the ASD diagnosis in adulthood. This is important in order to differentiate between a late emergence of any noticeable symptoms on the one hand *versus* misdiagnosis (i.e., ASD symptoms have not or not sufficiently been detected) or overshadowing (i.e., ADHD has been diagnosed instead of the correct diagnosis ASD) on the other hand. A recent study showed – similarly to some earlier studies – that a majority of those with an adult autism diagnosis had no records of having received a diagnosis in childhood (84% of males and 91% of females) or adolescence (69% of males and 61% of females) (52). The authors concluded that in the majority, i.e., cases with no psychiatric diagnosis in childhood, the late first diagnosis of ASD is unlikely to be explained by either misdiagnosis or overshadowing. This leads to the often raised question whether individuals first diagnosed with ASD and IQ above average as late as in adulthood differ substantially from individuals with a “prototypical” ASD diagnosis as a child-onset condition and profound developmental disorder (53).

To date, we do not know whether or not late diagnosed individuals with ASD are developmentally, phenomenologically, and biologically distinct from individuals diagnosed in childhood (52). From our perspective, two topics are worthy of further investigation in this context:

1. It is relevant to foster the development of more valid and more appropriate ASD diagnostic tools as well as external assessments in adult individuals with suspected ASD (48) especially if they have an additional *intellectual disability*. For example, as the standard behavioral observation diagnostic instrument [ADOS-2, Lord et al. (41)] for ASD has no appropriate module for adolescents and adults with intellectual disability, there is a strong need to (a) develop further valid and sensitive diagnostic instruments like the Diagnostic Behavioral Assessment for Autism Spectrum disorders-Revised [DiBAS-R (54, 55)] and (b) to recommend the application of these tools in individuals with intellectual disability in diagnostic guidelines.
2. Simultaneously, for adult individuals with an IQ above average and suspected ASD, better compensation strategies are assumed biasing the results of existing ASD diagnostic instruments. For example, they might be less prone to the situations created by the ADOS to detect ASD symptoms. Hence, more research is needed to (a) identify those symptoms which optimally discriminate between ASD and non-ASD cases and subsequently (b) to develop more sensitive diagnostic tools for this adult group. Hence, further research is needed whether adults with an above average IQ and symptoms of ASD should receive the diagnosis of ASD or rather another one, like for example, a personality disorder (as initially suggested by Asperger)?

In summary, the assumed interactions between age, IQ and ASD diagnosis are doubtless very complex resulting in the heterogeneity of individuals with ASD. This has the consequence that both sample characterization in studies and individual diagnostics is very challenging, and this in turn limits the interpretability and replicability of study results. In this context, we question with this perspective article whether existing ASD gold standard diagnostic tools were designed and validated for use in all these heterogeneous groups (particularly due to IQ). Therefore, a lot of research should be initiated (56) to develop, evaluate and implement ASD- symptomatology-, age- and IQ-related subtypes. This might help to grasp heterogeneity in order to increase validity and sensitivity of diagnostic instruments. This will only be possible with more longitudinal studies following large enough numbers of individuals with (suspected) ASD without IQ related in- or exclusion criteria; use of advanced technologies like machine learning to identity subtypes of ASD; identification of mechanisms of symptom change to develop personalized, evidence-based assessments and interventions.

## Data Availability Statement

The data analyzed in this study is subject to the following licenses/restrictions: Restrictions apply to the availability of the data (presented in Figure 1), which were used under license for the current study, and so are not publicly available. Requests to access these datasets should be directed to the NW, nicole.wolff@uniklinikum-dresden.de.

## Ethics Statement

The studies involving human participants were reviewed and approved by the local Ethics Committee (Az. 92/20). Written informed consent from the participants’ legal guardian/next of kin was not required to participate in this study in accordance with the National Legislation and the Institutional Requirements.

## Author Contributions

NW executed the study idea, prepared and analyzed the data, wrote the first draft of the manuscript, and incorporated the comments and remarks from the coauthors. SS, IK-B, and SR reviewed the manuscript, added comments, and rewrote parts of the manuscript. VR collaborated in all stages of the editing process of the final manuscript, added comments, and reviewed the manuscript from the first to the final draft. All authors contributed to the article and approved the submitted version.

## Conflict of Interest

The authors declare that the research was conducted in the absence of any commercial or financial relationships that could be construed as a potential conflict of interest.

## Publisher’s Note

All claims expressed in this article are solely those of the authors and do not necessarily represent those of their affiliated organizations, or those of the publisher, the editors and the reviewers. Any product that may be evaluated in this article, or claim that may be made by its manufacturer, is not guaranteed or endorsed by the publisher.
